# Rolling Resistance and Mechanical Properties of Grinded Copper Surfaces Using Molecular Dynamics Simulation

**DOI:** 10.1186/s11671-016-1616-1

**Published:** 2016-09-15

**Authors:** Shih-Wei Liang, Chih-Hao Wang, Te-Hua Fang

**Affiliations:** Department of Mechanical Engineering, National Kaohsiung University of Applied Sciences, Kaohsiung, 80778 Taiwan

**Keywords:** Grinding, Molecular dynamics, Rotation velocity, Rolling resistance

## Abstract

Mechanical properties of copper (Cu) film under grinding process were accomplished by molecular dynamics simulation. A numerical calculation was carried out to understand the distributions of atomic and slip vector inside the Cu films. In this study, the roller rotation velocity, temperature, and roller rotation direction change are investigated to clarify their effect on the deformation mechanism. The simulation results showed that the destruction of materials was increased proportionally to the roller rotation velocity. The machining process at higher temperature results in larger kinetic energy of atoms than lower temperature during the grinding process of the Cu films. The result also shows that the roller rotation in the counterclockwise direction had the better stability than the roller rotation in the clockwise direction due to significantly increased backfill atoms in the groove of the Cu film surface. Additionally, the effects of the rolling resistances on the Cu film surfaces during the grinding process are studied by the molecular dynamics simulation method.

## Background

The tribological and grinding characteristics of films on the nanoscale have become increasingly important due to increasing numbers of applications such as nanoimprint technology [1] and roller-type nanoimprint lithography (RNIL) [2]. Li et al. [3] used surface mechanical grinding treatment (SMGT) at cryogenic temperatures to synthesize a gradient nano-micro-structure in the surface layer of bulk metals. Fang et al. [4] used a SMGT for preparing a nanograined copper film with a spatial gradient in grain size and showed a different governing deformation mechanism.

Li et al. [5] reported that the mechanisms of subsurface damage and material removal of monocrystalline copper in nanoscale high-speed grinding and result showed that a large tip radius or depth of cut would get a greater temperature rise in the workpiece and lower grinding velocity made more intrinsic stacking faults. However, they more accurately evaluated the properties of the material by applying molecular dynamics (MD) simulations to the rolling–machining process, thereby incorporating more preliminary information for the tooling design and determining the optimum processing conditions. Wu et al. [6] studied the effect of the roller tooth’s taper angle, imprint depth, and imprint temperature on the properties of single-crystalline gold and observed that imprint force and adhesion increase with increasing imprint depth and decreasing taper angle. Lin et al. [7] used a MD simulation with the embedded atom method (EAM) to study the deformation process of pure copper nanorods in the nanoforming process; they reported that the pure copper nanorods undergo plastic deformation because of structural defects owing to higher energies in the material, wherein the higher energies are induced by large compressive loadings and high temperatures. Furthermore, the rolling process is similar to the milling, polishing, grinding, and cutting processes performed with a machining center. On the basis of MD simulations, Yang et al. [8] proposed a single-crystalline copper structure for ultra-precision polishing with the self-rotation of a diamond abrasive. They observed that an increase in abrasive rotation velocity decreased the tangential force, resulting in diminished material machine quality.

In a previous study [9], various rolling–sliding processes associated with a diamond in a Cu system were simulated by the MD method. The numerical results showed the maximum normal and frictional forces of Cu at a rotation velocity of zero in the rolling–sliding process because of the very high sliding resistance at the interface between the diamond and Cu. Unfortunately, the authors did not report the details of the rolling resistance for the Cu material surface. Thus, many challenges remain, such as understanding the rolling resistance, to fully elucidate the grinding mechanism of a roller on a Cu surface.

As evident from the above discussion, an MD-simulation-based, atomic-scale investigation of the mechanical properties and deformation mechanism of Cu films used in the grinding process with various roller rotation velocities is warranted. In the present study, we focus on the effects of the roller rotation velocity, temperature, direction, and rolling resistance on the grinding process of Cu films using MD simulations. The results are discussed in terms of the slip vector, deformation mechanism, and rolling resistance.

## Methodology

In our MD simulations, a three-dimensional physical system is used to describe the mechanical properties of Cu films during grinding processes. The rolling nanoimprint process is applied to face-centered cubic Cu films. For the MD simulation, the Cu film is initially assumed to have a well-defined atomic surface. The roller was set as the rigid body in the simulation, and the radius *r* of the diamond structure was 3.0 nm. The rollers comprise 15,083 carbon atoms, and the film comprises 37,842 Cu atoms. The dimensions of the Cu film substrate are 15.0 (length) × 3.0 (width) × 9.5 (height) nm^3^; they include a 1-nm fixed layer and a 1-nm thermostat layer in the right and bottom sides. The size of the film and the roller that has been selected for these simulations was thin enough from the previous film thickness affecting the results and has been used previously in several similar studies [10]. The roller feeding rate (along the *Y*-direction) and depth of the grinding process were set at 30 m/s and 1.5 nm, respectively. The traditional high-speed cutting speed was about 1–3 m/s in the cutting direction. The cutting speed of the ultra-speed diamond machine for copper was about 480~1200 m/min. Previous MD studies have selected the high-speed rate to save the computation time. In addition, the previous study [11] showed no direct effect on the cutting force in the range of 50–200 m/s. The high speed of grinding has been selected for these simulations, because it is computationally inexpensive, and it has been used for ultra-high-speed machining processes. We expect it to be adequate for qualitative investigation of the grinding phenomena. The initial distance between the roller and the film was set as 2.0 nm. A periodic boundary condition [12] was imposed on the *X*-axis of the surface plane, and the *Y*- and *Z*-axes were set to the real specimen height. The NTV ensemble (constant number *N* of particles, temperature *T*, and volume *V*) was used in this study.

In this study, the atomic interactions between C and Cu are described using the many-body potential via tight-binding second-moment approximation, because it has been demonstrated to be more accurate than the EAM potential and is simple to apply in MD simulations [13, 14]. The Morse potential model is used to describe the atomic interactions between C and Cu. The time integration of motion is constructed using Gear’s fifth-order predictor–corrector method, with a time step of 1 fs; the Verlet neighbor list method is used to increase the calculation efficiency.

## Results and Discussion

### Analysis of Grinding Simulation in the Cu Films

**Fig. 1:**
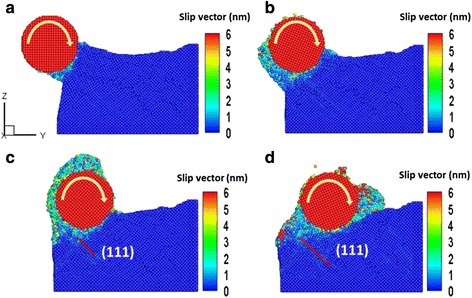
Variation of the slip vector and deformation mechanism of the Cu films during the rolling nanoimprint process at machining distances of **a** 1 nm, **b** 3 nm, **c** 5 nm, and **d** 8 nm

To clearly describe the strain distribution in deformed material, we determined the slip vector of a Cu film in this study using the difference in atomic positions between the initial and specific time steps after thermal equilibrium. The variations of the slip vector and deformation mechanism of the Cu films during the rolling nanoimprint process at machining distances of (a) 1, (b) 3, (c) 5, and (d) 8 nm are shown in Fig. 1. In the simulation, the operating temperature was 298 K, with a roller rotation rate of 10°/ps during the nanoimprint process. At a roller machining distance (*D*) of 1 nm, as shown in Fig. 1a, the surface atoms of the Cu films collide over almost the whole roller contact region and cause extensive destruction of the material structure. As evident in Fig. 1b, at *D* = 3 nm, an adsorption phenomenon occurs on the roller surface via extrusion induced by the rotation force. The ground chips piled up around the roller. As shown in Fig. 1c, d, downward pressure continued to increase significantly with increasing *D* as the roller rotated, thereby causing the Cu film to gradually deform and create a new slip plane along the <110> direction [15].

### Effect of Roller Rotation Velocity

**Fig. 2:**
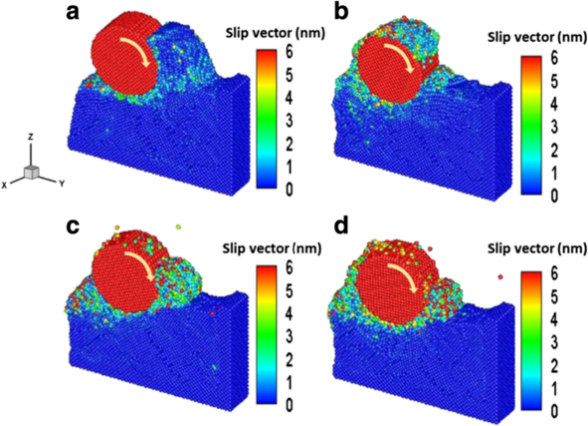
Variation of slip vector distributions of Cu film at a machining distance of 8 nm for roller rotation velocities of **a** 0°/ps, **b** 5°/ps, **c** 10°/ps, and **d** 20°/ps

We used MD simulations to study the influence of roll and scratch processing on Cu films formed at 298 K. Figure 2 shows the slip vector distributions of Cu films at a machining distance of 8 nm for roller rotation velocities of (a) 0°/ps, (b) 5°/ps, (c) 10°/ps, and (d) 20°/ps, respectively. At a roller rotation velocity of 0°/ps (pure scratching), the dislocations were observed to nucleate and propagate preferentially along the feed direction of the roller on the Cu film surface; only small changes in the slip vector and no significant phenomenon of backfilling in the Cu film were observed after the scratching process. We also observed that the material removal mechanism is dominated by plowing, as indicated by the removed atoms accumulating in front of the roller during the rolling nanoimprint process. The slip amount of Cu films increases with roller rotation velocity, because the atomic bonding strength in the roller feeding with velocity direction is reduced. The scratching process results in shorter and curled chips and lower friction heat compared with the rolling nanoimprint process; the scratching process thus leads to lower roller wear.

**Fig. 3:**
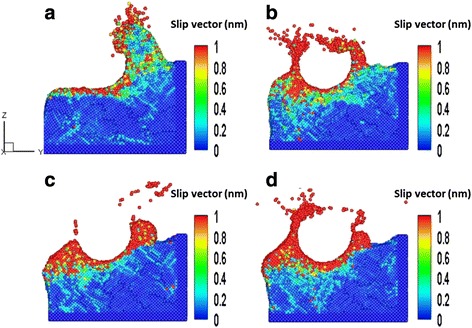
Snapshots of the rolling nanoimprint process of the side-view atomic flow field of Cu films at the roller rotation velocities of **a** 0°/ps, **b** 5°/ps, **c** 10°/ps, and **d** 20°/ps, respectively

Figure 3 shows the snapshots of the side-view atomic flow field during the grinding process at the roller rotation velocities of (a) 0°/ps, (b) 5°/ps, (c) 10°/ps, and (d) 20°/ps, respectively. Figure 3 clearly shows that a relationship exists between the atomic flow fields and the roller rotation velocity of the Cu film. The effect of rotary pressure increases with the roller rotation velocity of the Cu film, and the atomic flow fields increase toward the *Y*-axis direction. This behavior is explained by the effect of the strain energy under the invasive action of the roller. Higher roller rotation velocity appears to increase the ability to remove atomic backfill onto the groove; conceivably, adhesion and rotational resistance between the removed atoms and the roller could substantially affect the removal mechanism of the atoms.

**Fig. 4:**
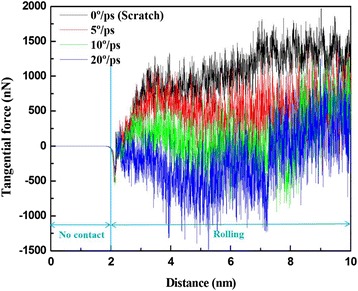
Variation of tangential force Fy with roller machining distance for Cu film with roller rotation velocities of 0, 5, 10, and 20°/ps, respectively

Figure 4 shows the tangential force Fy as a function of machining distance for Cu films with different roller rotation velocities of (a) 0°/ps, (b) 5°/ps, (c) 10°/ps, and (d) 20°/ps. As evident in this figure, the tangential force of the Cu films at higher roller rotation velocities is enhanced compared with that of the Cu films at lower roller rotation velocities, as indicated by the tangential force of the Cu film at higher roller rotation velocities exhibiting a large increase in strain rate during the grinding process. However, the tangential force was also increased, because more chips pile up in front of the roller at higher roller rotation velocities than at lower roller rotation velocities. These results are in agreement with those previously reported by Cui et al. [16].

### Effect of Rolling Temperature

**Fig. 5:**
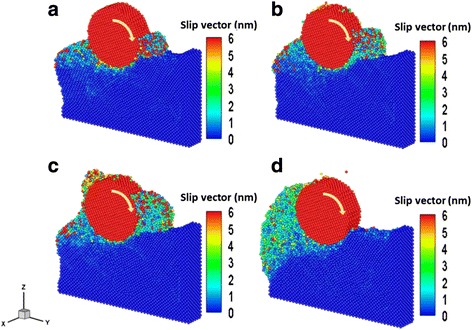
Snapshots of the slip vector and deformation mechanism of the Cu films at a machining distance of 8 nm for temperatures of **a** 150 K, **b** 300 K, **c** 450 K, and **d** 750 K, respectively

In this section, the rolling nanoimprint of Cu film properties for various temperatures is calculated to investigate the effect of atom–atom interactions and nanoforming behavior. The roller rotation velocity and roller machining distance were 20°/ps and 8 nm, respectively. Figure 5 shows the snapshots of the slip vector and deformation mechanism of the Cu films at temperatures of (a) 150 K, (b) 300 K, (c) 450 K, and (d) 750 K, respectively. The results indicate that the kinetic energy of the atoms generally increases with system temperature during the grinding process; this increase in kinetic energy is a consequence of the increased activity among the atoms at elevated system temperatures, which results in a material softening phenomenon. This effect was also reported by Wu et al. [17].

**Fig. 6:**
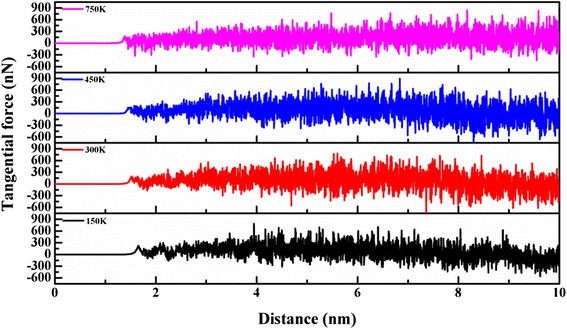
Variation of tangential force Fy with roller machining distance for temperatures of 150, 300, 450, and 750 K, respectively

Figure 6 shows the tangential force Fy as a function of roller machining distance for Cu films at temperatures of 150, 300, 450, and 750 K. The simulation results for various temperatures indicate that the tangential force is effectively increased at higher temperatures because of the enhancement in van der Waals interactions between the indenter and the Cu film with increasing temperature [18]. However, the extent of material softness increases with temperature, which causes an increase in force feedback in the material during the roller machining process.

### Effect of Roller Rotation Direction

**Fig. 7:**
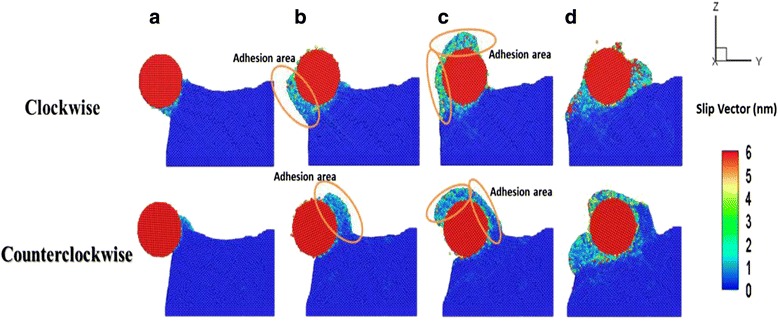
Variation of slip vector distributions of Cu film with various roller rotation directions at machining distances of **a** 1 nm, **b** 3 nm, **c** 5 nm, and **d** 8 nm

To investigate the effect of roller rotation direction on the grinding process, we simulated clockwise and counterclockwise roller rotation directions. The working temperature was set at 298 K, and the roller rotation velocity was set at 10 and −10°/ps with clockwise and counterclockwise roller rotation directions, respectively. Figure 7 shows the slip vector distributions of Cu films with various roller rotation directions at machining distances of (a) 1 nm, (b) 3 nm, (c) 5 nm, and (d) 8 nm, respectively. As shown in Fig. 7a, the adsorption forces between the roller and the Cu films’ surface atoms were increased under both clockwise and counterclockwise roller rotation directions, resulting in the increased destruction of the Cu films during the initial roller machining process. Figure 7b shows the film obtained at a roller machining distance *D* of 3 nm. With clockwise roller rotation direction, we observed that the Cu film atoms moved downward under extrusion, which caused an atom pile up on the side of Cu films. However, under counterclockwise rotation direction, the Cu film atoms moved upward and piled up on the roller to form a chip, because the Cu film was segregated from the substrate by a rotation roller. The accumulation of removed atoms around the roller increased with the roller machining distance for each film under both clockwise and counterclockwise roller rotation directions. This increased accumulation was due to the increase in the area of the adhesion region with increasing machining distance, as shown in Fig. 7c. As shown in Fig. 7d, with a clockwise roller rotation direction, the slip system along the <110> direction was generated and affected by the defects of the Cu film surface because of greater atom adhesion between the roller and the Cu films during the nanoimprint process. However, we observed no significant changes in the slip system along the <110> direction in the case of counterclockwise roller rotation.

**Fig. 8:**
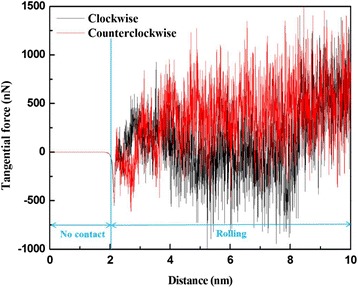
Variation of tangential force Fy with roller machining distance for roller rotation direction of clockwise and counterclockwise

Figure 8 shows the tangential force Fy as a function of the machining distance for Cu films subjected to clockwise and counterclockwise roller rotation. In the simulation, the roller does not yet contact the Cu film at a roller machining distance of 0–2 nm (along the *Y*-direction), followed by rolling at a roller machining distance of 2–10 nm. As also shown in Fig. 8, the Fy slightly increases with machining distance under clockwise roller rotation during the machining process. This behavior is due to backfilled atoms on the path of the machining process causing increased resistance in the roller; it is also associated with the evolution of the dislocations in the material. In addition, the Fy of the roller rotation in the counterclockwise direction appears to be more stable than that of the roller rotation in the clockwise direction. This greater stability is due to the substantial increase in the number of backfill atoms in the groove of the Cu film surface.

### Rolling Resistance

**Fig. 9:**
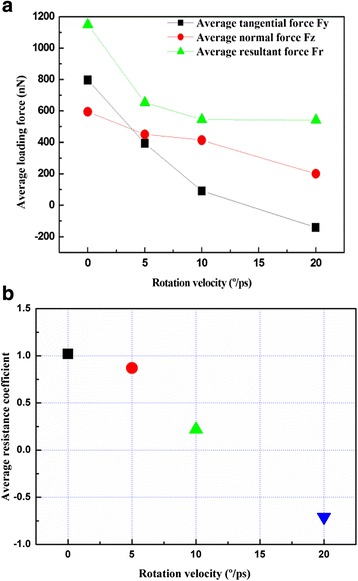
Variation of **a** average loading force and **b** average resistance coefficient with roller rotation velocity at a temperature of 300 K

To evaluate the rolling resistance of the Cu film surfaces at various roller rotation velocities and rolling temperatures during the grinding process, the average rolling resistance coefficient *μ*_*R*_ of the Cu film surface is defined by the following equation:

1 $$ {\mu}_R=\frac{F_y}{F_z}, $$

where *F*_*y*_ is the average tangential force and *F*_*z*_ is the average normal force at contact between the roller and the Cu film surfaces during the rolling process. The average loading force and average resistance coefficient for various roller rotation velocities at 300 K and a roller machining distance of 3 nm are shown in Fig. 9a, b, respectively. As the roller rotation velocity increases from 0 to 20°/ps, the average loading force and resistance coefficient of the Cu films decrease; this result was expected because of the increased influence of rotation resistance between the roller and the Cu film interface. This observation is in good agreement with previous nanometric cutting simulation reports [19, 20]. Interestingly, *F*_*y*_ is greater than *F*_*z*_ during the scratching process, thereby indicating that the average resistance coefficient was slightly greater than 1.0, as shown in Fig. 9b. This difference between *F*_*y*_ and *F*_*z*_ could be due to the adhesive force field that exists between the roller and the Cu film, which results in an increased stick–slip behavior. These results are in good agreement with previous literature reports [21, 22]. Moreover, the average resistance coefficient decreased to negative values at a roller rotation velocity of 20°/ps, probably because the average tangential force exhibits a negative value, and thus, the resistance force tends toward the sliding direction [21, 23].

**Fig. 10:**
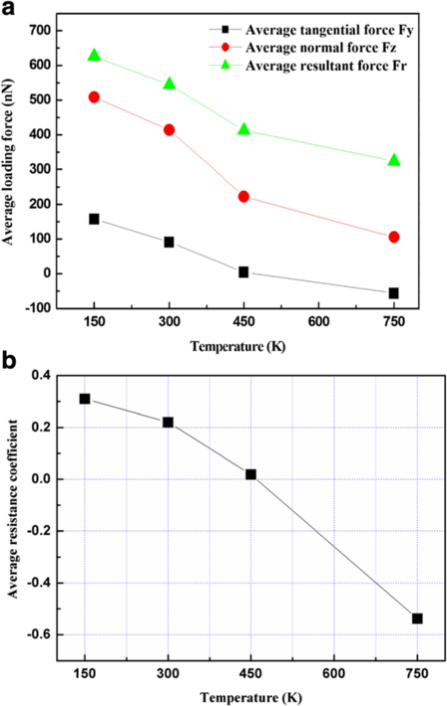
Variation of **a** average loading force and **b** average resistance coefficient with temperature at a roller rotation velocity of 10°/ps

The average loading force and average resistance coefficient for various temperatures at a roller rotation velocity of 10°/ps and roller machining distance of 3 nm are shown in Fig. 10a, b, respectively. As evident in the figure, the average loading force and resistance coefficient of Cu films decreased with increasing temperature from 150 to 750 K. The reason for this behavior is that increases in the number of airborne atoms result in a greater increase in material softening and crystalline structure disordering [24]. However, a negative average resistance coefficient was observed when the temperature was increased to 750 K, probably as a consequence of carbon atom diffusion, leading to the low hardness of the Cu film surface.

## Conclusion

In this study, we investigated in detail the mechanical properties under a grinding process of Cu films using MD simulations. Our conclusions are as follows:

1. The downward pressure of the roller loading on the Cu films increased with *D*, thus resulting in an increased deformation and the creation of a new slip plane.
2. The kinetic energy and bond strength of atoms increased with temperature because of higher activity among the atoms. The temperature variation strongly affected the mechanical properties of the Cu films.
3. Counterclockwise roller rotation was more stable than clockwise roller rotation, because the amount of backfill atoms in the groove of the Cu film surface substantially increased.
4. In the case of clockwise roller rotation, a slip system was generated along the <110> direction because of increased atomic adhesion between the roller and the Cu films during the grinding process.
5. When the temperature was increased, the average loading force and resistance coefficient of Cu films decreased, because the number of airborne atoms increased, thus resulting in increased material softening and increased disordering of the crystalline structure.
